# The impact of PA/I38 substitutions and PA polymorphisms on the susceptibility of zoonotic influenza A viruses to baloxavir

**DOI:** 10.1007/s00705-023-05958-5

**Published:** 2024-01-12

**Authors:** Keiichi Taniguchi, Takeshi Noshi, Shinya Omoto, Akihiko Sato, Takao Shishido, Keita Matsuno, Masatoshi Okamatsu, Scott Krauss, Richard J Webby, Yoshihiro Sakoda, Hiroshi Kida

**Affiliations:** 1 Shionogi Pharmaceutical Research Center, Shionogi & Co., Ltd., Toyonaka, Osaka Japan; 2 Department of Disease Control, Faculty of Veterinary Medicine, Hokkaido University, Sapporo, Hokkaido Japan; 3 International Institute for Zoonosis Control, Hokkaido University, Sapporo, Hokkaido Japan; 4 Institute for Vaccine Research and Development, HU-IVReD, Hokkaido University, Sapporo, Hokkaido Japan; 5 International Collaboration Unit, International Institute for Zoonosis Control, Hokkaido University, Sapporo, Hokkaido Japan; 6 One Health Research Center, Hokkaido University, Sapporo, Hokkaido Japan; 7 St. Jude Children’s Research Hospital, Memphis, TN United States

## Abstract

Genetic reassortment of avian, swine, and human influenza A viruses (IAVs) poses potential pandemic risks. Surveillance is important for influenza pandemic preparedness, but the susceptibility of zoonotic IAVs to the cap-dependent endonuclease inhibitor baloxavir acid (BXA) has not been thoroughly researched. Although an amino acid substitution at position 38 in the polymerase acidic protein (PA/I38) in seasonal IAVs reduces BXA susceptibility, PA polymorphisms at position 38 are rarely seen in zoonotic IAVs. Here, we examined the impact of PA/I38 substitutions on the BXA susceptibility of recombinant A(H5N1) viruses. PA mutants that harbored I38T, F, and M were 48.2-, 24.0-, and 15.5-fold less susceptible, respectively, to BXA than wild-type A(H5N1) but were susceptible to the neuraminidase inhibitor oseltamivir acid and the RNA polymerase inhibitor favipiravir. PA mutants exhibited significantly impaired replicative fitness in Madin-Darby canine kidney cells at 24 h postinfection. In addition, in order to investigate new genetic markers for BXA susceptibility, we screened geographically and temporally distinct IAVs isolated worldwide from birds and pigs. The results showed that BXA exhibited antiviral activity against avian and swine viruses with similar levels to seasonal isolates. All viruses tested in the study lacked the PA/I38 substitution and were susceptible to BXA. Isolates harboring amino acid polymorphisms at positions 20, 24, and 37, which have been implicated in the binding of BXA to the PA endonuclease domain, were also susceptible to BXA. These results suggest that monitoring of the PA/I38 substitution in animal-derived influenza viruses is important for preparedness against zoonotic influenza virus outbreaks.

**Supplementary Information:**

The online version contains supplementary material available at 10.1007/s00705-023-05958-5.

## Introduction

Influenza A viruses (IAVs) exhibit zoonotic potential to infect both birds and mammals (e.g., pigs and humans) [1, 2]. IAVs remain a persistent health threat due to the presence of populations of quasispecies [3–5] attributable to the extraordinarily high mutation rate of these viruses [6–8]. One of the more striking evolutionary features of IAVs is genetic reassortment, which leads to the emergence of pandemic-causing viruses [9, 10]. IAVs maintained in aquatic wild birds can infect humans during epidemics in poultry and livestock, when they efficiently adapt and replicate because the segmented RNA genome of IAVs readily allows for genetic reassortment, thus facilitating the emergence of new viruses [11]. Direct transmission of IAVs from animals to humans also poses a significant threat to human health due to potentially high morbidity and mortality [e.g., A(H5N1) and A(H7N9)] [12–14]. Sporadic outbreaks of infection caused by various subtypes [e.g., A(H3N8), A(H5N8), A(H9N2), A(H10N3)] have been reported over the last few years [15–18]. The Centers for Disease Control (CDC) has recommended treatment with anti-influenza virus drugs for human cases of avian influenza virus infection [19], and neuraminidase inhibitors (NAIs) such as oseltamivir and zanamivir have been used to treat patients infected with H5 and H7 influenza viruses [20, 21]. However, drug-resistant mutants [e.g., NA/H275Y of A(H5N1) or NA/R292K of A(H7N9) at N2 numbering] have emerged after NAI treatment in some cases [22, 23]. The same amino acid substitutions have also been detected in seasonal influenza viruses [24, 25].

Baloxavir marboxil (BXM), which is converted metabolically to its active form baloxavir acid (BXA), is an orally available cap-dependent endonuclease (CEN) inhibitor that has been approved for clinical use in adults and adolescents worldwide [26]. The influenza virus polymerase complex is composed of one polymerase acidic (PA) and two polymerase basic (PB1 and PB2) proteins [27]. BXA targets CEN located in the PA N-terminal domain, which is highly conserved among IAVs [28]. Variants exhibiting reduced susceptibility to BXA have been detected in some seasonal influenza patients who had received BXM therapy [28, 29]. The major amino acid substitution associated with reduced susceptibility to BXA among seasonal influenza viruses is an isoleucine-to-threonine substitution at amino acid position 38 in the PA N-terminal domain (PA/I38T), although isoleucine-to-phenylalanine or -methionine substitutions (PA/I38F or M) can also occur [30, 31]. Several studies have examined the impact of the PA/I38 substitution on the fitness of various seasonal influenza virus strains [31–34]. However, many characteristics of zoonotic influenza viruses remain unclear because natural polymorphisms at this residue are rare [35, 36], and unlike the case of NAIs, genetic markers of BXA susceptibility in zoonotic influenza viruses have not been clearly identified.

In the present study, we evaluated the BXA susceptibility of recombinant A(H5N1) viruses harboring single PA/I38F, M, or T substitutions in addition to various avian and swine strains with PA polymorphisms. In order to assess the impact of PA/I38 substitutions on BXA susceptibility and replicative fitness, recombinant A/Hong Kong/483/97 (H5N1) strains harboring individual substitutions were generated. We previously characterized the virus harboring the NA-H275Y substitution for an *in vitro* BXA efficacy study [37]; therefore, this strain was used to verify BXA, oseltamivir acid (OSA), and favipiravir (FPV) susceptibility and cross-resistance.

## Materials and methods

### Compounds

BXA was synthesized at Shionogi & Co., Ltd. OSA was purchased from Toronto Research Chemicals Inc. (Toronto, Ontario, Canada). FPV was supplied by PharmaBlock Sciences, Inc. (Nanjing, China).

### Cells and viruses

Madin-Darby canine kidney (MDCK) cells (European Collection of Cell Cultures) were maintained at 37°C under 5% CO_2_ in minimum essential medium (MEM; Nissui Pharmaceutical) supplemented with 10% heat-inactivated fetal bovine serum, 2 mM L-glutamine, 50 units of penicillin and 50 µg of streptomycin per mL, and 0.05% sodium hydrogen carbonate. Recombinant A/Hong Kong/483/1997 (H5N1) viruses [the wild-type (WT) virus and viruses harboring PA/I38F, M, and T substitutions] were generated and propagated as described previously [37]. The avian and swine IAVs tested in this study (28 strains in total) were selected considering isolation areas, subtypes, separation dates, and PA amino acid polymorphisms (Online Resource 1). These viruses were propagated in embryonated chicken eggs and harvested from virus-containing allantoic fluids. Infectivity titers were determined by standard 50% tissue culture infectious dose (TCID_50_) assays in MDCK cells. Virus titers were calculated based on the visible virus-induced cytopathic effect (CPE) and expressed as log_10_ TCID_50_/mL. The amino acid sequences in the PA N-terminal region of recombinant A/Hong Kong/483/1997 (H5N1) viruses and other avian or swine IAVs tested in this study were predicted by Sanger sequencing of the corresponding region of the genome (Online Resource 1). Briefly, viral RNA obtained from allantoic fluids was extracted using a QIAamp Viral RNA Mini Kit (QIAGEN) according to the manufacturer’s protocol. Reverse transcription, amplification of cDNA, and sequencing were performed as reported previously [38]. The following primers were used in this study: forward, 5′-GCAGGTACTGATCCGAAATG-3′; reverse, 5′-GGAGAAGTTAGGTGGGAGAC-3′. The region encoding the PA N-terminal domain was sequenced by the Sanger method at Eurofins Genomics (Tokyo, Japan). The deduced amino acid sequences of the PA proteins of the avian and swine IAVs tested in this study were submitted to the National Center for Biotechnology Information (NCBI) database or the Global Initiative on Sharing All Influenza Data (GISAID), and their accession numbers are listed in Online Resource 1.

### Virus yield reduction assay

Virus yield reduction assays were performed as described previously [37, 39]. Briefly, MDCK cells (30,000 cells/well) pre-seeded in 96-well plates were infected with each virus at 100 TCID_50_/well and then incubated at 35°C under 5% CO_2_ for 1 h. The virus inoculum was removed by washing, and fresh MEM with or without (recombinant H5N1 viruses only) acetylated trypsin (final concentration: 0.0025 mg/mL) and defined concentrations of test compounds were added to the infected cells. BXA and FPV were dissolved in dimethyl sulfoxide (DMSO), and OSA was dissolved in distilled water (DW). The diluted solutions had a final concentration of 0.5% DMSO or 0.5% DW. As untreated controls, fresh MEM with or without acetylated trypsin, DMSO, and DW were used (final concentration: 0.5% each). The cells were incubated at 35°C under 5% CO_2_ for 24 h, and viral titers in the culture supernatants were determined by TCID_50_ assay in MDCK cells. Virus titers were calculated based on the visible virus-induced CPE and expressed as log_10_ TCID_50_/mL. The 90% effective concentration (EC_90_) values were calculated as the concentration necessary to decrease the viral titer in the culture supernatant to one-tenth of that of the untreated control, using a linear interpolation method. The mean and standard deviation (SD) were calculated from three independent experiments.

### Genetic analysis

PA nucleotide sequences for human, avian, and swine influenza viruses collected between January 1, 2012, and September 21, 2022 (total: 41,537) were obtained from NCBI and GISAID on September 21, 2022, and aligned using GENETYX® ver. 14.0 for Windows (GENETYX Corp., Japan).

### Evaluation of virus replicative fitness

MDCK cells (30,000 cells/well) were seeded in 96-well plates 1 day prior to infection. Cells in each well were infected with 100 TCID_50_ of the recombinant virus. The infected cells were then incubated at 35°C under 5% CO_2_ for 1 h and washed with MEM, followed by the addition of fresh MEM and further incubation at 35°C under 5% CO_2_. Cell culture supernatants were collected at the indicated time points, and virus titers (log_10_ TCID_50_/mL) were determined by TCID_50_ assay in MDCK cells. Virus titers were calculated based on the visible virus-induced CPE and expressed as log_10_ TCID_50_/mL.

### Statistical analysis

Differences in titer between the WT virus and mutants harboring the PA/I38F, M, or T substitution were examined at each time point using Welch’s *t*-test. Statistical analysis was conducted using the statistical analysis software SAS, version 9.4 for Windows (SAS Institute, Cary, NC, USA). A *p*-value < 0.05 was considered statistically significant.

## Results

### BXA susceptibility and replicative fitness of recombinant A/Hong Kong/483/97 (H5N1) with variations in PA

**Fig. 1:**
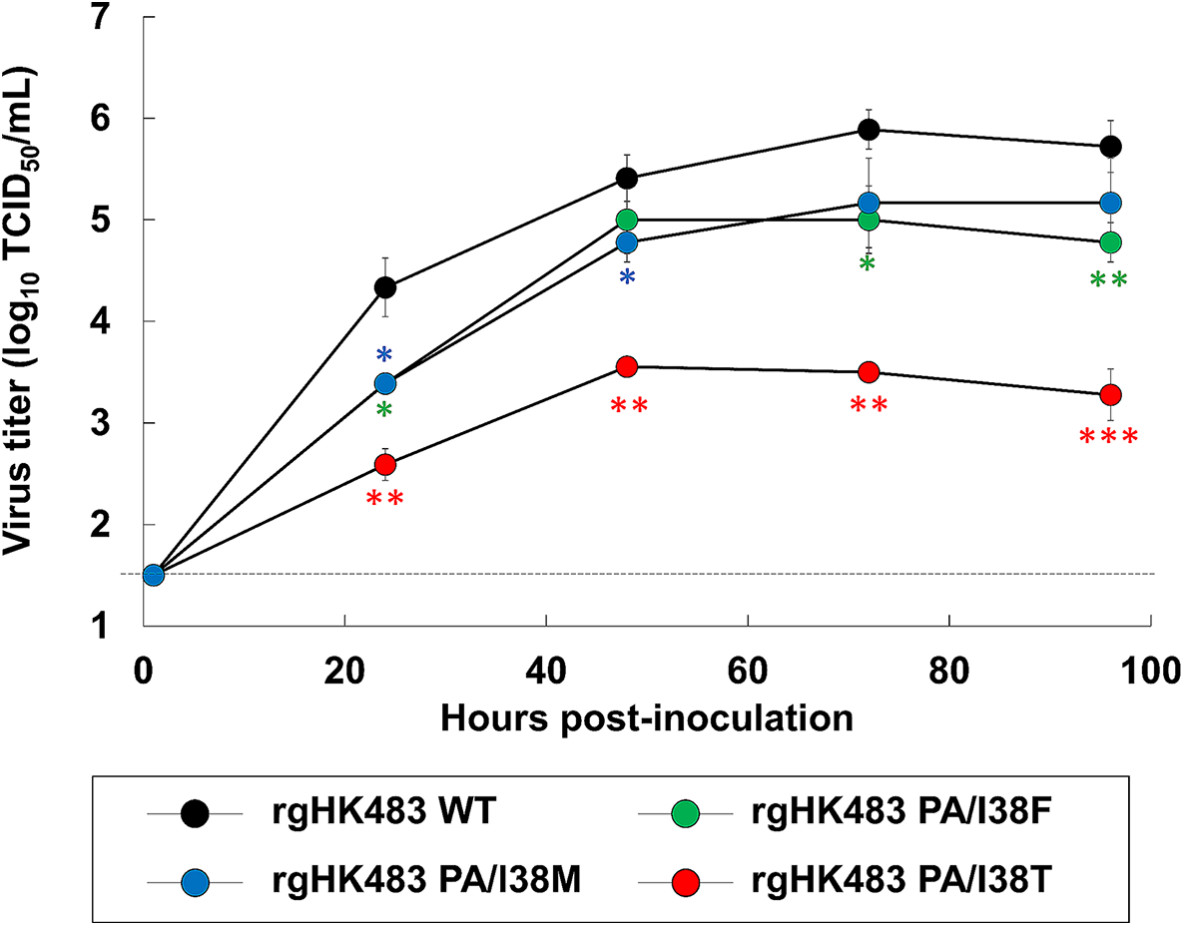
In vitro replicative fitness of recombinant A/Hong Kong/483/97 (H5N1) viruses. MDCK cells were infected with recombinant viruses at 100 TCID_50_/well. Supernatants were harvested at the indicated time points, and the mean virus titers of triplicate wells ± SD of the mean were determined as TCID_50_/mL using MDCK cells. The lower limit of quantification (1.5 log_10_ TCID_50_/mL) of the virus titer is indicated by a dashed line. HK483, A/Hong/Kong/483/97 (H5N1); WT, wild type. Welch’s *t*-test was used for statistical comparisons of titers between the WT virus and viruses with PA/I38F, M, and T substitutions at each time point (*, *p* < 0.05; **, *p* < 0.01; ***, *p* < 0.001 compared to WT virus)

In order to assess the impact of PA/I38 substitutions on BXA susceptibility and replicative fitness, recombinant A/Hong Kong/483/97 (H5N1) strains harboring individual substitutions were generated, and their susceptibility to BXA and replicative fitness were tested in MDCK cells. Compared to the recombinant WT virus, which exhibited a mean EC_90_ value of 1.1 nM, the PA/I38F, M, and T variants exhibited 24.0-, 15.5-, and 48.2-fold higher EC_90_ values, respectively (Table 1). By contrast, OSA and FPV showed comparable inhibitory activity against each virus. The replicative capacity of each PA-substituted virus was significantly lower than that of the WT virus in MDCK cells at 24 h postinfection, and each virus replicated to lower titers at all time points compared to the WT virus (Fig. 1). These data indicate that PA mutants, especially the PA/I38T strain, exhibit significantly lower BXA susceptibility and impaired fitness compared to the WT virus. The PA mutants were susceptible to OSA and FPV, which have different mechanisms of action from BXA. In a previous study, BXA exhibited antiviral activity against recombinant A/Hong Kong/483/97 (H5N1) containing an NA/H275Y substitution [37].

**Table 1:** Susceptibility of recombinant A/Hong Kong/483/97 (H5N1) viruses harboring PA amino acid substitutions to BXA, oseltamivir acid, and favipiravir

	EC_90_ (nmol/L)								
	BXA		Fold change	Oseltamivir acid		Fold change	Favipiravir		Fold change
Strain	Mean	SD		Mean	SD		Mean	SD	
rgA/Hong Kong/483/97 (H5N1) WT	1.1	0.5	NA	17.3	6.3	NA	10,409.7	1,826.6	NA
rgA/Hong Kong/483/97 (H5N1) PA/I38F	26.5	13.9	24.0	29.2	13.4	1.7	16,252.7	11,840.9	1.6
rgA/Hong Kong/483/97 (H5N1) PA/I38M	17.1	4.7	15.5	30.3	11.7	1.7	12,698.5	7,518.7	1.2
rgA/Hong Kong/483/97 (H5N1) PA/I38T	53.3	28.6	48.2	21.6	17.7	1.2	12,699.6	4,722.9	1.2

Data represent the mean and standard deviation (SD) from three independent experiments

WT, wild type; NA, not applicable

### BXA susceptibility of temporally and geographically distinct avian and swine influenza viruses

**Fig. 2:**
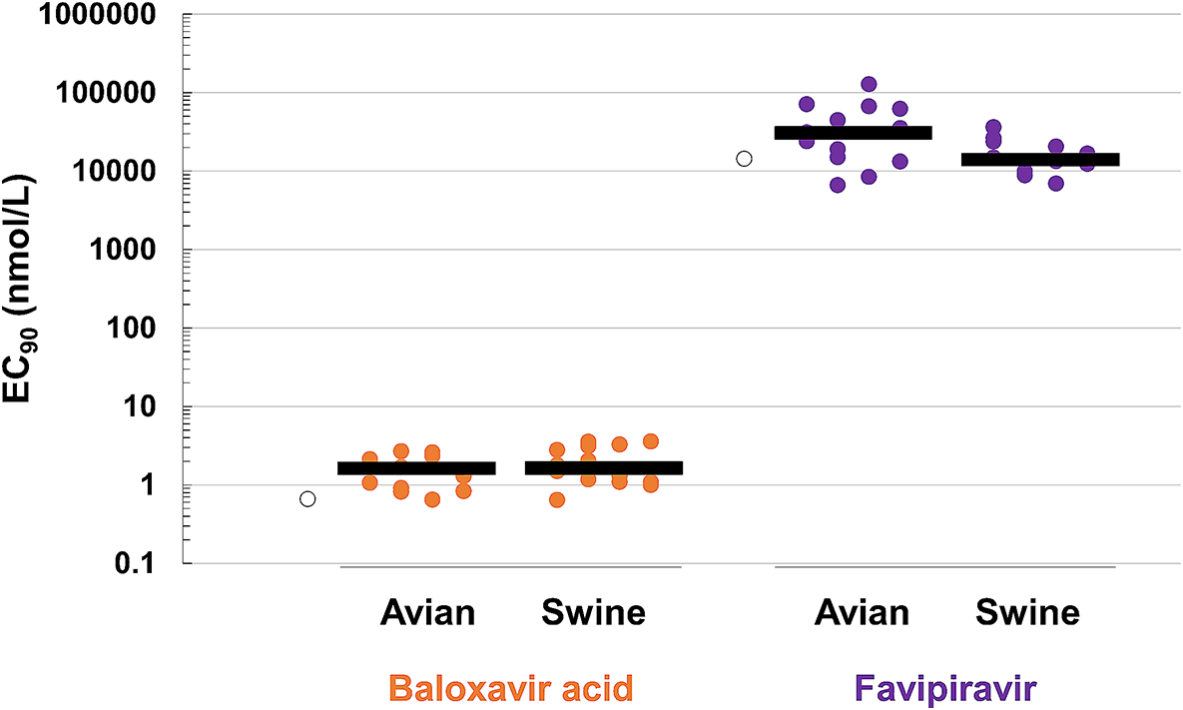
Susceptibility of temporally and geographically distinct avian and swine influenza viruses to BXA in a virus yield reduction assay using MDCK cells. Subtypes of avian and swine viruses differing by year and country of isolation were subjected to virus yield reduction assays with BXA and favipiravir. Data are represented as scatter plots with combined EC_90_ values. White circles indicate the antiviral activity of BXA (EC_90_ = 0.7 ± 0.4 nmol/L) or favipiravir (EC_90_ = 14368.0 ± 11855.7 nmol/L) against the A/Puerto Rico/8/34 (H1N1) strain as a reference

Previously, we reported that zoonotic influenza viruses of subtypes H1, H5, H7, and H9 were susceptible to BXA *in vitro*, similar to human clinical isolates of subtypes H1 and H3 [32, 37, 39]. For a thorough characterization of the spectrum of BXA activity, drug susceptibility testing was performed against avian and swine strains (28 strains in total). The median EC_90_ value of BXA was 1.6 nM for both avian and swine strains (Fig. 2 and Online Resource 1). Among the PA amino acid polymorphisms, PA/I38 variants were rare, whereas A20T, Y24H, and A37S were present in more than 1% of all of the viruses whose sequences were obtained from the database and analyzed (Table 2). The amino acid polymorphisms A20T, Y24H, and A37S, which have been implicated in the binding of BXA to the PA endonuclease domain [28], did not impact BXA susceptibility (Table 2). The median EC_90_ values of FPV were 30,433.5 nM and 13,957.1 nM for avian and swine strains, respectively. These data indicate that the tested viruses, which varied in terms of isolation area, subtype, date of isolation, and PA amino acid polymorphisms, were susceptible to BXA at levels comparable to those of previously reported IAVs [32, 37, 39].

**Table 2:** Amino acid polymorphisms in the BXA binding domain of PA from avian and swine influenza viruses

	PA amino acid position^a^												
Influenza virus strain	20	23	24	34	37	38	41	80	108	119	130	134	199
A/Puerto Rico/8/34 (H1N1)	**T**	E	Y	K	A	I	H	E	D	E	Y	K	E
A/chicken/Vietnam/HU1-381/2014 (H9N2)	A	E	Y	K	**S**	I	H	E	D	E	Y	K	E
A/swine/Okinawa/2/2005 (H1N1)	**T**	E	Y	K	A	I	H	E	D	E	Y	K	E
A/swine/Miyagi/5/2003 (H1N2)	**T**	E	Y	K	A	I	H	E	D	E	Y	K	E
A/swine/Miyazaki/1/2006 (H1N2)	**T**	E	Y	K	A	I	H	E	D	E	Y	K	E
A/swine/Ratchaburi/2000 (H1N1)	A	E	**H**	K	A	I	H	E	D	E	Y	K	E
A/swine/Chachoengsao/2002 (H3N2)	A	E	**H**	K	A	I	H	E	D	E	Y	K	E
Other viruses evaluated	A	E	Y	K	A	I	H	E	D	E	Y	K	E
Human, avian, and swine influenza A viruses isolated^b^	A (94.7)	E (100)	Y (98.0)	K (99.9)	A (92.5)	I^c^ (99.5)	H (100)	E (100)	D (100)	E (100)	Y (100)	K (100)	E (99.5)
	T (5.2)		H (2.0)		S (7.4)								

^a^ The indicated amino acids were previously shown to be involved in binding of BXA to the active center of the endonuclease domain in the PA subunit (residues 20, 24, 34, 37, 38, 41, 80, 108, 119, 130, and 134) and associated with reduced susceptibility to BXA (residues 23, 37, 38, and 199) [28]. Amino acids differing from the consensus sequence of human influenza A viruses are highlighted in bold and underlined. Consensus sequences were determined by alignment with the full-length PA sequences of human, avian, and swine influenza viruses collected between January 1, 2012, and September 21, 2022, obtained from NCBI and GISAID on September 21, 2022.

^b^ Numbers shown in parentheses represent the frequency (%) of the most common variants among PA sequences from the analyzed viruses (total: 41,537).

^c^ The frequencies of the variants were as follows: V, 0.41%; L, 0.02%; T, 0.02%; M, 0.01%.

## Discussion

Several reports have shown that PA/I38F, M, and T variant viruses isolated from BXM-treated patients exhibit reduced susceptibility to BXA [31, 40]. Natural variants harboring PA polymorphisms, such as PA/I38M, I38L, and E23G, exhibit 4- to 10-fold reduced BXA susceptibility at low frequencies [35]. A few natural occurrences of amino acid polymorphisms such as PA/E199G, A36T, and I38V have been reported [41]. These polymorphisms have been suggested to play a role in the binding of BXA to the PA endonuclease domain [28]. However, examination of sequence databases revealed that PA/I38 substitutions in isolates from animals are rare, and therefore, the BXA susceptibility of these isolates has not been determined. As a result, genetic markers of reduced susceptibility to BXA in zoonotic viruses have not been clearly identified. Recently, the susceptibility of A(H5N1) viruses harboring PA/I38T, I38M, and A37T to BXA was reported to be similar to that of seasonal influenza viruses [36]. However, the effect of a single PA/I38 substitution on the replicative fitness of other zoonotic influenza viruses is still unknown. In the present study, the impact of three major PA/I38 substitutions (I38T, F, and M) on BXA susceptibility was examined using recombinant A(H5N1) viruses. The recombinant A/Hong Kong/483/97 (H5N1) isolates harboring PA/I38F, M, and T substitutions showed lower BXA susceptibility, and the degree of reduction in BXA susceptibility was comparable to that of seasonal viruses, as reported previously [28, 42, 43]. Previously, it was reported that the PA/I38T substitution in seasonal A/H1N1pdm09 virus was predicted to cause structural changes of the active site of the PA endonuclease domain that weaken BXA binding, resulting in reduced BXA susceptibility [28]. Notably, A(H5N1) and seasonal A(H1N1pdm) and A(H3N2) viruses exhibited similar X-ray crystal structures of the CEN active site and surrounding amino acids [27, 44]. These data support our findings regarding decreased BXA susceptibility in PA/I38-substituted seasonal IAVs and A/Hong Kong/483/97 (H5N1) strains. The PA amino acid sequences of other zoonotic viruses [e.g., A(H7N9)] are similar to that of A(H5N1) [39]; therefore, PA/I38 substitutions could be potential genetic markers for BXA susceptibility of zoonotic viruses.

Seasonal H1 or H3 viruses with PA/I38 mutations, especially PA/I38F or T, exhibit reduced fitness [28, 42, 43] in MDCK cells, whereas variants with PA/I38M or T substitutions exhibit fitness comparable to that of the WT virus [40, 45]. Our results show that, compared to the WT virus, the PA/I38T mutant had the most significantly impaired fitness, whereas in zoonotic IAVs, the PA/I38F and PA/I38M mutants tend to exhibit impaired fitness. These observations suggest that H5 viruses harboring PA/I38 substitutions are less fit than seasonal strains. The PA/I38 substitution has been associated with impaired CEN activity in influenza A and B viruses [28], suggesting that the CEN activity of A(H5N1) with PA/I38 substitutions was impaired, resulting in decreased viral growth. Similar observations have been made with NAIs. Resistant H5 or H7 influenza viruses have been isolated after treatment of human patients with NAIs [22, 23]. The mutations detected in those cases were NA/H275Y for A(H5N1) and NA/R292K for A(H7N9). The positions of these NA amino acid substitutions and the susceptibility of these viruses to OSA were similar to those of seasonal IAVs [22, 23]. In this study, only one recombinant strain of an animal-derived influenza virus was evaluated; thus, it will be necessary to examine the impact of naturally occurring PA/I38 substitutions and other PA polymorphisms in primary cells derived from humans and birds as described previously [36]. Previous studies have shown that seasonal influenza A viruses (IAVs) with the PA/I38 substitution have lower replicative capacity in MDCK cells than the wild type [28, 42, 43]. On the other hand, PA mutants of seasonal IAVs tend to have lower [34, 45, 46] or similar [31, 40, 42] replicative capacity in mice or hamsters than the wild type. It is therefore expected that the *in vivo* replicative capacity of the recombinant A(H5N1) viruses is similar to or lower than that of the wild type. However, there are other strain-specific differences in replication capacity *in vivo.* Therefore, *in vivo* studies of the infectivity and transmissibility of PA mutants are needed.

Previous reports indicated that several IAVs isolated from animals were susceptible to BXA [32, 47, 48], but to date, there are no reports of polymorphisms associated with decreased BXA susceptibility. To address this issue, we evaluated the BXA susceptibility of avian and swine strains harboring various PA polymorphisms, isolated primarily in North and South America, Europe, and Asia, with differing subtypes and separation dates. All of the tested strains were susceptible to BXA, and therefore, no amino acid polymorphisms associated with reduced BXA susceptibility were identified in the study. A genetic analysis of the amino acid residues involved in the binding of BXA to the PA endonuclease domain [28] conducted over the course of a decade revealed that the amino acid polymorphisms PA/A20T, Y24H, and A37S were present in > 1% of isolates (Table 2). These PA polymorphisms were not associated with BXA susceptibility, but more studies are needed to evaluate other existing polymorphisms, such as those at positions 34 and 199. Accumulating evidence suggests that I38 substitutions in the PA proteins of IAVs, including zoonotic viruses, have the greatest impact on reducing the susceptibility to BXA [28, 35, 49, 50, 51].

In conclusion, data from phenotypic analysis suggest that BXA exhibits broad-spectrum antiviral activity against a wide range of circulating IAVs in birds and pigs. Although PA/I38 is highly conserved among recently isolated IAVs, continuous monitoring of PA polymorphisms (including those involving I38) in animal-derived influenza viruses is needed.

### Electronic Supplementary Material

Below is the link to the electronic supplementary material

Supplementary Material 1
