# Supplementary material for: The impact of PA/I38 substitutions and PA polymorphisms on the susceptibility of zoonotic influenza A viruses to baloxavir
Source: Arch Virol. 2024 Jan 12;169(2):29. doi: 10.1007/s00705-023-05958-5 (PMC10786730; doi:10.1007/s00705-023-05958-5)
Supplement: Supplementary file 1 — Supplementary Material 1 [file 705_2023_5958_MOESM1_ESM.pdf]

The impact of PA/I38 substitution and PA polymorphisms on susceptibility of zoonotic influenza A viruses to baloxavir

Archives of Virology

Keiichi Taniguchi[1, 2], Takeshi Noshi[1], Shinya Omoto[1], Akihiko Sato[1, 3], Takao Shishido[1, *], Keita Matsuno[3, 4, 5, 6], Masatoshi Okamatsu[2], Scott Krauss[7], Richard J Webby[7], Yoshihiro Sakoda[2, 4, 5, 6] and Hiroshi Kida[3, 4, 5]

[1]Shionogi & Co., Ltd., Osaka, Japan. [2]Department of Disease Control, Faculty of Veterinary Medicine, Hokkaido University, Japan. [3]International Institute for Zoonosis Control, Hokkaido University, Sapporo, Japan. [4]Institute for Vaccine Research and Development, HU-IVReD, Hokkaido University, Sapporo, Japan. [5]International Collaboration Unit, International Institute for Zoonosis Control, Hokkaido University, Sapporo, Japan. [6]One Health Research Center, Hokkaido University, Sapporo, Japan. [7]Department of Infectious Diseases, St. Jude Children's Research Hospital, United States.

[*]To whom correspondence should be addressed: Dr. Takao Shishido, Shionogi Pharmaceutical Research Center, 3-1-1, Futaba-cho, Toyonaka, Osaka 561-0825, Japan; Tel: +81-6-6331-7263, E-mail: takao.shishido@shionogi.co.jp

Supplementary Figure 1. The sequencing chromatograms of PA mutation site in each recombinant A/Hong Kong/483/1997 (H5N1) virus harboring PA/I38T, F and M.

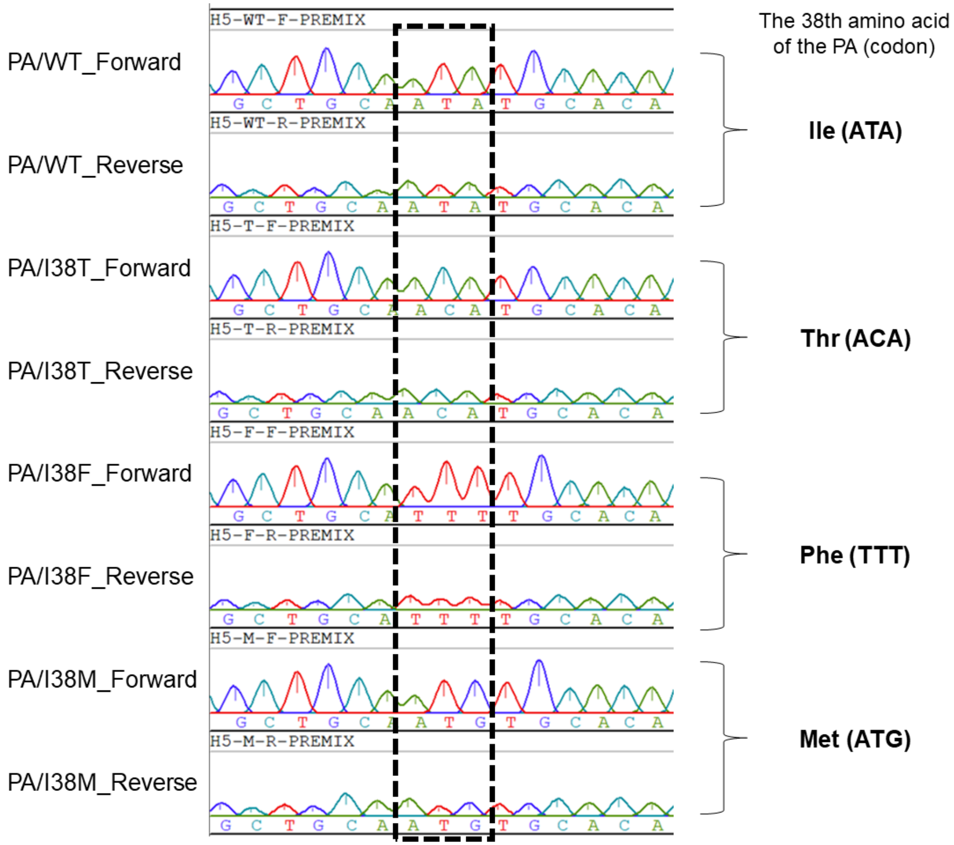

The amino acid sequences in the PA N-terminal region of recombinant A/Hong Kong/483/1997 (H5N1) viruses were determined by sanger sequence method. The mutation site, the 38th amino acid of the PA (dotted frame), was focused on this figure. Each PA sequence of all samples except above was completely matched with that of the reference (the parent virus).

Supplementary Table 1. The variants of PA region in viral RNAs derived from each virus stock.

a. Avian influenza viruses

Strain	Accession number	Nucleic acid				Amino acid			
		Position	Reference	Call	Minor peak	Position	Reference	Call	Minor peak
A/yellow-billed pintail/Chile/1/2012 (H1N1)	CY207202*	97	A	A	T	33	N (AAT)	N (AAT)	Y (TAT)
A/red-fronted coot/Chile/5/2013 (H3N6)	CY207027*	294	T	T	C	98	T (ACT)	T (ACT)	T (ACC)
A/pekin duck/California/P30/2006 (H4N2)	CY053826*	-	-	-	-	-	-	-	-
A/shorebird/Delaware Bay/309/2008 (H4N6)	CY126603*	111	A	A	C	37	A (GCA)	A (GCA)	A (GCC)
A/duck/Hokkaido/WZ20/2014 (H5N2) [#]	LC042072*	N.T.	N.T.	N.T.	N.T.	N.T.	N.T.	N.T.	N.T.
A/ruddy turnstone/Delaware Bay/136/2007 (H6N1)	CY127788*	-	-	-	-	-	-	-	-
A/duck/Yamagata/061004/2014 (H6N6) [#]	LC042080*	N.T.	N.T.	N.T.	N.T.	N.T.	N.T.	N.T.	N.T.
A/laughing gull/Delaware Bay/50/2006 (H7N3)	CY095653*	-	-	-	-	-	-	-	-
A/yellow-billed pintail/Chile/10/2014 (H7N3)	CY207226*	87	A	A	G	29	K (AAA)	K (AAA)	K (AAG)
A/yellow-billed teal/Chile/9/2013 (H7N6)	CY207034*	-	-	-	-	-	-	-	-
A/chicken/Netherlands/2586/2003 (H7N7)	AB438940*	-	-	-	-	-	-	-	-
A/chicken/Vietnam/HU1-381/2014 (H9N2) [#]	LC069901*	N.T.	N.T.	N.T.	N.T.	N.T.	N.T.	N.T.	N.T.
A/shorebird/Delaware Bay/139/2009 (H10N7)	CY137799*	-	-	-	-	-	-	-	-
A/shorebird/Delaware Bay/549/2009 (H11N1)	CY127892*	111	A	A	C	37	A (GCA)	A (GCA)	A (GCC)

The amino acid sequence in the PA N-terminal region including amino acids at position 20 to 199, involved in binding of BXA to the active center of the endonuclease

domain in the PA subunit and associated with reduced susceptibility to BXA [28]. Amino acids differing from the reference sequence of each tested virus are highlighted in underlined. The accession numbers listed in Supplementary Table 1 and reference sequences of each tested virus were obtained from NCBI (*). -: PA sequence was completely matched with that of the reference sequence. #: Sequence analysis was not performed as the sequence had already been determined. Each PA sequence of all samples except above was completely matched with that of the reference (the parent virus). N.T.: not tested.

b. Swine influenza viruses

Strain	Accession number	Nucleic acid				Amino acid			
		Position	Reference	Call	Minor peak	Position	Reference	Call	Minor peak
A/swine/Iowa/15/1930 (H1N1)	M26076*	75	A	G	-	25	G (GGA)	G (GGG)	-
		134	C	G	-	45	S (TCC)	C (TGC)	-
		172	G	A	-	58	G (GGC)	S (AGC)	-
		253	G	A	-	85	A (GCA)	T (ACA)	-
		366	T	A	-	122	V (GTT)	E (GAA)	-
		435	T	C	-	145	I (ATT)	I (ATC)	-
		519	C	T	-	173	T (ACC)	T (ACT)	-
		576	C	T	-	192	R (CGC)	R (CGT)	-
A/swine/Kagoshima/23/2012 (H1N1)	AB910570*	-	-	-	-	-	-	-	-
A/swine/Okinawa/2/2005 (H1N1)	AB573799*	-	-	-	-	-	-	-	-
A/swine/Ratchaburi/2000 (H1N1) [#]	AB434287*	N.T.	N.T.	N.T.	N.T.	N.T.	N.T.	N.T.	N.T.
A/swine/Miyagi/5/2003 (H1N2)	LC431443*	-	-	-	-	-	-	-	-
A/swine/Miyazaki/1/2006 (H1N2)	AB441175*	-	-	-	-	-	-	-	-
A/swine/Missouri/2124514/2006 (H2N3)	EU258940*	-	-	-	-	-	-	-	-
A/swine/Chachoengsao/2002 (H3N2)	AB571802*	-	-	-	-	-	-	-	-

b. Swine influenza viruses (continued)

Strain	Accession number	Nucleic acid				Amino acid			
		Position	Reference	Call	Minor peak	Position	Reference	Call	Minor peak
A/swine/Hong Kong/81/1978 (H3N2)	LC431427*	87	A	A	T	29	K (AAA)	K (AAA)	N (AAT)
		180	A	A	C	60	S (TCA)	S (TCA)	S (TCC)
A/swine/Obihiro/10/1985 (H3N2)	AB573672*	-	-	-	-	-	-	-	-
A/swine/Tochigi/14/2013 (H3N2)	AB914507*	-	-	-	-	-	-	-	-
A/swine/Yokohama/aq114/2011 (H3N2)	AB741022*	-	-	-	-	-	-	-	-
A/swine/Hong Kong/9/1998 (H9N2)	KX879588*	-	-	-	-	-	-	-	-
A/swine/Hong Kong/10/1998 (H9N2)	EPI2077974**	-	-	-	-	-	-	-	-

The amino acid sequence in the PA N-terminal region including amino acids at position 20 to 199, involved in binding of BXA to the active center of the endonuclease domain in the PA subunit and associated with reduced susceptibility to BXA [28]. Amino acids differing from the reference sequence of each tested virus are highlighted in underlined. The accession numbers listed in Supplementary Table 1 and reference sequences of each tested virus were obtained from NCBI (*) or GISAID (**). -: PA sequence was completely matched with that of the reference sequence. #: Sequence analysis was not performed as the sequence had already been determined. Each PA sequence of all samples except above was completely matched with that of the reference (the parent virus). N.T.: not tested.

Supplementary Table 2. EC_{90} values of baloxavir acid and favipiravir against the tested viruses in yield reduction assays using MDCK cells.

a. Avian influenza viruses

		EC_{90} (nmol/L)			
		Baloxavir acid		Favipiravir	
Strain	Isolation area	Mean	SD	Mean	SD
A/yellow-billed pintail/Chile/1/2012 (H1N1)	Chile	2.1	2.1	31205.3	28039.2
A/red-fronted coot/Chile/5/2013 (H3N6)	Chile	2.0	1.1	23846.5	17398.6
A/pekin duck/California/P30/2006 (H4N2)	US	2.1	1.2	8459.1	3869.7
A/shorebird/Delaware Bay/309/2008 (H4N6)	US	1.1	0.9	29661.6	32384.2
A/duck/Hokkaido/WZ20/2014 (H5N2)	Japan	0.9	0.8	14931.3	11108.6
A/ruddy turnstone/Delaware Bay/136/2007 (H6N1)	US	0.8	0.7	6627.2	3008.5
A/duck/Yamagata/061004/2014 (H6N6)	Japan	2.7	2.6	18912.6	11886.1
A/laughing gull/Delaware Bay/50/2006 (H7N3)	US	0.6	0.3	13179.8	2983.0
A/yellow-billed pintail/Chile/10/2014 (H7N3)	Chile	2.6	2.0	71284.8	71632.8
A/yellow-billed teal/Chile/9/2013 (H7N6)	Chile	2.3	1.1	66809.9	57540.6
A/chicken/Netherlands/2586/2003 (H7N7)	Netherlands	1.7	0.3	127541.1	92368.6
A/chicken/Vietnam/HU1-381/2014 (H9N2)	Vietnam	0.8	0.5	62342.3	58578.3
A/shorebird/Delaware Bay/139/2009 (H10N7)	US	1.5	0.7	35234.0	15776.7
A/shorebird/Delaware Bay/549/2009 (H11N1)	US	1.3	0.4	44721.8	39518.3

Data represent the mean and standard deviation (SD) from three independent experiments.

b. Swine influenza viruses

Strain	Isolation area	EC_{90} (nmol/L)			
		Baloxavir acid		Favipiravir	
		Mean	SD	Mean	SD
A/swine/Iowa/15/1930 (H1N1)	US	2.8	1.6	6907.6	1888.2
A/swine/Kagoshima/23/2012 (H1N1)	Japan	1.5	0.8	26428.2	16669.8
A/swine/Okinawa/2/2005 (H1N1)	Japan	1.8	1.2	36110.5	36400.0
A/swine/Ratchaburi/2000 (H1N1)	Thailand	3.6	1.8	23645.9	34236.0
A/swine/Miyagi/5/2003 (H1N2)	Japan	3.1	3.0	9954.1	5718.4
A/swine/Miyazaki/1/2006 (H1N2)	Japan	1.2	0.4	13480.9	12250.7
A/swine/Missouri/2124514/2006 (H2N3)	US	2.0	1.0	8822.4	4888.9
A/swine/Chachoengsao/2002 (H3N2)	Thailand	3.5	2.2	13363.6	10734.1
A/swine/Hong Kong/81/1978 (H3N2)	China	1.3	1.1	14826.5	11814.6
A/swine/Obihiro/10/1985 (H3N2)	Japan	3.3	1.7	20563.6	8091.6
A/swine/Tochigi/14/2013 (H3N2)	Japan	1.1	0.5	13386.4	13356.3
A/swine/Yokohama/aq114/2011 (H3N2)	Japan	1.1	0.8	16773.1	17235.5
A/swine/Hong Kong/9/1998 (H9N2)	China	1.0	0.6	12296.8	6689.8
A/swine/Hong Kong/10/1998 (H9N2)	China	0.6	0.1	14433.2	12669.8

Data represent the mean and standard deviation (SD) from three independent experiments.
